# The effect of hippocampal damage in children on recalling the past and imagining new experiences

**DOI:** 10.1016/j.neuropsychologia.2011.03.008

**Published:** 2011-06

**Authors:** Janine M. Cooper, Faraneh Vargha-Khadem, David G. Gadian, Eleanor A. Maguire

**Affiliations:** a Developmental Cognitive Neuroscience Unit, Institute of Child Health, University College London, 30 Guilford Street, London WC1N 1EH, United Kingdom; b Radiology and Physics Unit, Institute of Child Health, University College London, 30 Guilford Street, London WC1N 1EH, United Kingdom; c Wellcome Trust Centre for Neuroimaging, Institute of Neurology, University College London, 12 Queen Square, London WC1N 3BG, United Kingdom

**Keywords:** Autobiographical memory, Scene construction, Episodic memory, Hippocampus, Children, Developmental amnesia

## Abstract

Compared to adults, relatively little is known about autobiographical memory and the ability to imagine fictitious and future scenarios in school-aged children, despite the importance of these functions for development and subsequent independent living. Even less is understood about the effect of early hippocampal damage on children's memory and imagination abilities. To bridge this gap, we devised a novel naturalistic autobiographical memory task that enabled us to formally assess the memory for recent autobiographical experiences in healthy school-aged children. Contemporaneous with the autobiographical memories being formed, the children also imagined and described fictitious scenarios. Having established the performance of healthy school-aged children on these tasks, we proceeded to make comparisons with children (*n* = 21) who had experienced neonatal hypoxia/ischaemia, and consequent bilateral hippocampal damage. Our results showed that healthy children could recall autobiographical events, including spatiotemporal information and specific episodic details. By contrast, children who had experienced neonatal hypoxia/ischaemia had impaired recall, with the specific details of episodes being lost. Despite this significant memory deficit they were able to construct fictitious scenarios. This is in clear contrast to adults with hippocampal damage, who typically have impaired autobiographical memory and deficits in the construction of fictitious and future scenarios. We speculate that the paediatric patients’ relatively intact semantic memory and/or some functionality in their residual hippocampi may underpin their scene construction ability.

## Highlights

► Children with bilateral hippocampal damage are impaired at recalling past events. ► They are intact at imagining fictitious experiences. ► This contrasts with adult amnesic patients who are impaired at both. ► Hippocampal damage affects memory differently depending on when in life it occurs.

## 1 Introduction

Being able to recall the past and imagine and plan for the future are prerequisites for independent living. The hippocampus is part of a network of brain regions that supports the retrieval of autobiographical memories (Cabeza & St. Jacques, 2007; Maguire, 2001; Spreng, Mar, & Kim, 2009; Svoboda, McKinnon, & Levine, 2006). Functional MRI (fMRI) findings have indicated the hippocampus is also involved in imagining fictitious episodes (Hassabis, Kumaran, & Maguire, 2007a; Summerfield, Hassabis, & Maguire, 2009, 2010), and the simulation of personal future events (e.g. Addis & Schacter, 2008; Addis, Wong, & Schacter, 2007; Addis, Pan, Vu, Laiser, & Schacter, 2009; Botzung, Denkova, & Manning, 2008; Okuda et al., 2003; Szpunar, Watson, & McDermott, 2007). Further neuropsychological evidence for this comes from Hassabis, Kumaran, Vann, and Maguire (2007b), (see also Andelman, Hoofien, Goldberg, Aizenstein, & Neufeld, 2010; Klein, Loftus, & Kihlstrom, 2002; Rosenbaum et al., 2005; but see Squire et al., 2010, and Maguire & Hassabis, 2011 for a response) who tested patients with bilateral hippocampal damage that was acquired in adulthood, rendering them amnesic. They were asked to imagine and describe fictitious scenarios and also possible plausible future episodes. The patient group was significantly impaired relative to control participants on both tasks, and a possible source for their deficit was identified. While patients were able to produce relevant details when asked to imagine, their scene descriptions lacked spatial coherence and were fragmented. It was concluded that the hippocampus may play a critical role in imagination by binding together the disparate elements of an event or scene in a process called scene construction (Cohen and Eichenbaum, 1993; Hassabis & Maguire, 2007, 2009; Hassabis et al., 2007b; O’Keefe & Nadel, 1978; see Buckner & Carroll, 2007; Schacter & Addis, 2007 for other views).

Whilst Hassabis et al. (2007b) documented impairment of scene construction associated with adult-onset amnesia and bilateral hippocampal damage, it was recently reported that another patient with selective hippocampal lesions and developmental amnesia (DA; Vargha-Khadem et al., 1997) was unimpaired on the scene construction task (Maguire, Vargha-Khadem, & Hassabis, 2010). Jon, now an adult, sustained his hippocampal pathology perinatally, and showed the symptoms of DA from early childhood. His ability to describe detailed fictitious and future scenarios stands in marked contrast to the patients whose hippocampal damage and amnesia were acquired in adulthood (Hassabis et al., 2007b). Maguire et al. (2010) noted that Jon's intact scene construction could be due to the effortful strategies he had developed over the years to assist with imagination, perhaps relying on his well-developed semantic memory store, reflecting plastic changes in response to the neonatal hypoxic/ischaemic insults which may have ameliorated some of the effects of early hippocampal damage. Also, Jon's preserved scene construction could be associated with residual hippocampal function, given that his remaining hippocampal tissue activated during fMRI when he recalled the few autobiographical memories that he retains (Maguire, Vargha-Khadem, & Mishkin, 2001). By contrast another, now adult, DA patient HC (Case E6 in Vargha-Khadem et al., 2003; DA6 in Adlam, Vargha_Khadem, Mishkin, & de Haan, 2005; Adlam, Malloy, Mishkin, & Vargha-Khadem, 2009) was recently reported to be impaired at simulating personal future events (Kwan et al., 2010). The test used differed somewhat from that employed by Hassabis et al. (2007b) and Maguire et al. (2010), in that there were fewer trials, there was no spatial coherence measure, and the focus was on internal and external details – a legacy of the test's origins in the autobiographical memory literature. We suggest that this test may not be the most appropriate means of assessing the subtleties of imagination/simulation in patients. To examine this assertion directly, a study is currently underway in which patient HC's performance on the Hassabis et al. (2007b) test is being investigated (E.A. Maguire personal communication).

Given the uncertain status of scene construction/simulation in the context of single case studies of patients with DA, a large sample of children with neonatal hypoxia/ischaemia and associated hippocampal damage and memory impairment is required to evaluate the contributions of age and experience to processes involved in scene construction. In order to assess how children with hippocampal damage perform on autobiographical memory and scene construction tasks, it is necessary to compare such a patient group to healthy control children.

Surprisingly, studies of autobiographical memory in school-aged children and adolescents are fewer than those in adults. Bauer, Burch, Scholin, and Güler (2007) suggest that children between the ages of 7 and 10 years give mainly gist or fact-based information when recalling personal events and often do not provide details pertaining to who was present, or the spatiotemporal context of an event. Developmental changes to regions of the brain such as the frontal lobes, areas associated with retrieval abilities and executive functions in adolescence (Huizinga, Dolan, & van der Molen, 2006; Lehto, Juujärvi, Kooistra, & Pulkkinen, 2003), may account for the increase in contextual information observed (Pasupathi & Wainryb, 2010; Piolino et al., 2007) in older children's narratives of autobiographical events, an ability that has been found to continue to develop along with self-awareness through adolescence and into early adulthood (McLean, 2005; Pasupathi & Wainryb, 2010; for a review see Piolino, Desgranges, & Eustache, 2009). Picard, Reffuveille, Eustache, and Piolino (2010) also suggested the development of episodic event memory is linked to an increased efficiency of cognitive abilities, although little is known about other processes, such as scene construction, that may be involved. Thus, the relatively limited literature suggests that school-aged children and adolescents are able to recall everyday memories. While work is starting to emerge on future-oriented thinking in pre-schoolers (e.g. Atance & Jackson, 2009; Atance & Meltoff, 2006; Grant & Suddendorf, 2010; Suddendorf, Nielsen, & von Gehlen, 2011) much less has been documented formally about the ability of school-aged children and adolescents to construct imagined scenes and scenarios.

We therefore set out to examine a number of key issues. First, we devised a novel autobiographical memory task that enabled us to formally assess memory for recent autobiographical experiences in a group (*n* = 12) of healthy school-aged children. This ecologically valid task involved spending the day with each child during which a series of (unbeknownst to them) pre-planned ‘events’ occurred, memory for which was assessed on the subsequent day in a surprise recall test. In this way, each child underwent the same controlled yet naturalistic protocol to examine autobiographical recall, and our novel analysis method enabled different aspects of autobiographical recollection to be documented. Contemporaneous with the autobiographical memories being formed, the children also imagined and described fictitious scenarios in an adaptation of the Hassabis et al. (2007b) paradigm. Having recorded performance on the autobiographical memory recall and scene construction tests in healthy school-aged children, and adding to the literature thereon, we proceeded to compare the performance of the young healthy controls to that of a group of children who had been exposed to neonatal hypoxia/ischaemia with concomitant selective bilateral hippocampal volume loss, the majority of whom had memory problems. We report data from the largest group of such patients yet identified (*n* = 21) allowing us to establish with confidence the boundaries within which their autobiographical memory recall and scene construction abilities operate.

We hypothesised that the healthy control school-aged children would perform with high accuracy on the autobiographical memory retrieval test, and that they would provide rich descriptions of imagined scenarios. Given their hippocampal pathology, impairments on standard memory tests, and reports from parents of everyday memory problems, we expected the patient group to be impaired in autobiographical memory recall, similar to patients with adult-acquired selective hippocampal damage having resulted in amnesia. Regarding scene construction, the question was whether they would show the same intact profile as DA patient Jon, or resemble the impaired adult patients with amnesia?

## 2 Materials and methods

### 2.1 Participants

Twenty-one patients (13 male; mean age 13.29 years; SD 2.49; range 9–18 years; 3 left handed) participated, each of whom had suffered neonatal hypoxia/ischaemia due to cardiorespiratory disease. They were all born at term, had no neurodevelopmental abnormalities, were free of genetic syndromes, and were native English speakers. Twelve healthy control participants (6 male; mean age 12.33 years; SD 2.06; range 9–15 years; 2 left handed) also took part. All participants and their parents gave informed written consent to participate in the study in accordance with the regulations of the local research ethics committees. There was no significant difference between the groups in terms of age (*t* = −1.12, *P* = 0.27). Parental occupation was classified according to the International Standard Classification of Occupation (ISCO-88) and then transformed into an International Socio-Economic Index of Occupational Status, which provides socio-economic values for parental occupations. There was no significant difference between the patients and controls for socioeconomic status (*t* = 1.27, *P* = 0.22).

**Fig. 1:**
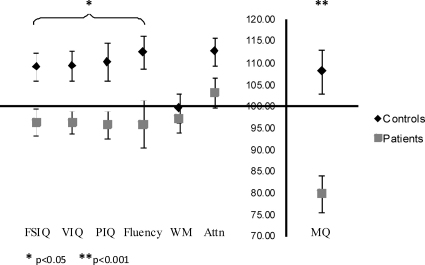
Mean scores (±1 standard error) for the controls and patients on baseline neuropsychological measures. Comparisons of mean scores between the controls and patients showed a significant difference, with the controls consistently scoring higher than the patients, although the magnitude of this difference was far greater in the domain of every day memory. Of note, the patients did not differ significantly from the population mean (100) of these standardised tests on any measure except every day memory (*P* < 0.0001). FSIQ = full scale IQ, VIQ = verbal IQ; PIQ = performance IQ; WM = working memory; Attn = attention; MQ = memory quotient (see text for details of the test instruments).

All participants were assessed for verbal, nonverbal and overall intelligence (Verbal IQ, Performance IQ, Full Scale IQ; Wechsler Intelligence Scale for Children 4th Ed.), verbal fluency (subtest of the Delis-Kaplan Executive Function System), and every day memory, working memory and attention (General Memory, Working Memory, Attention/Concentration; Children's Memory Scale) (see Fig. 1). The scores of the control group were at the high end of the average range (i.e. from 8 to 13 points above the standard mean of 100), and consequently higher than the scores of the patients which were within the average range and only 3–5 points below the standard population mean. When FSIQ was controlled for, there were no differences between the two groups on any measure except on every day memory (see Fig. 1); this was also the case when verbal and performance IQs were examined separately. Of note, neither age nor IQ correlated significantly with any of the experimental measures of recall and scene construction. This accords with previous use of the scene construction test (Hassabis et al., 2007), where background participant characteristics such as age and IQ did not affect scores on this task.

**Fig. 2:**
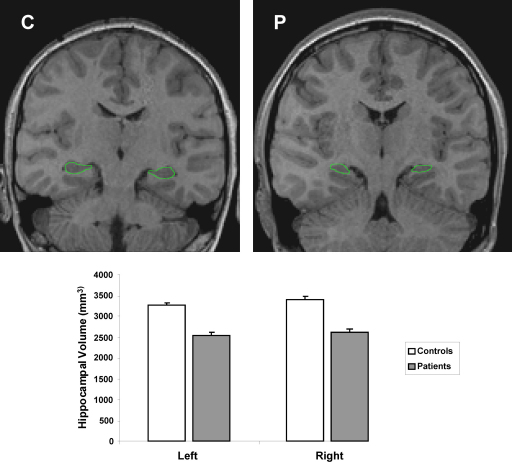
The top panels show MRI T1 images from an example control participant (C) and an age and gender matched patient (P), with the hippocampi outlined in green. The lower panel shows the mean (±standard error) left and right hippocampal volumes for control and patient groups.

Whole brain structural MRI scans were obtained for each participant using a 1.5T Siemens Avanto Scanner, with a T1-weighted 3D FLASH sequence with the following parameters: repetition time 11 ms, echo time 4.94 ms, flip angle 15°; matrix size 224 × 256; in-plane resolution 1 mm × 1 mm; partition thickness 1 mm; 176 sagittal partitions in the third dimension; acquisition time 5.34 min; no gap. The scans were examined by an experienced paediatric neuroradiologist to screen for detectable signal abnormality, space occupying lesions, or volume loss (other than hippocampal) across the whole brain. No consistent abnormalities outside of the medial temporal lobe were found. For the measurement of hippocampal volumes, the data were reformatted into 1 mm-thick contiguous slices in a tilted coronal plane perpendicular to the long axis of the hippocampus. Hippocampal cross-sectional areas were measured in all slices along the entire length of the hippocampus, using MEDx 3.43 (Medical Numerics, Inc., Maryland, USA) software. The volumes were calculated by summing the cross-sectional areas and multiplying by the distance between the slices. Corrections, derived from the regression line of control hippocampal volume versus intracranial volume, were then made for intracranial volume. Bilateral damage to the hippocampi was confirmed for the patient group (see Fig. 2), with a mean hippocampal volume bilaterally of 2550 mm^3^ (SD 386), compared to 3339 mm^3^ (SD 213) in the control group, a 21% reduction (*t* = 5.50, *P* = 0.001).

### 2.2 Tasks and procedures

Testing took place over two days. On day 1, each participant took part in events that (unbeknownst to them) followed a scripted protocol. There were 13 events in total, 10 that were used for the main experiment, and 3 for practice trials during recall. The events (e.g. choosing a snack, drawing a complicated picture) were found during pilot testing to fit seamlessly into a day of neuropsychological assessments. Piloting also revealed through participant ratings that all events were similar in their level of difficulty and memorability, as well as ensuring they were not emotive or obscure. Using discrete but thorough notes taken during the events, the experimenter chose the 10 events that contained full, clear and detailed actions and interactions to use in the memory test on day 2. All participants were unaware they would be asked to recall the events on the following day, or of the experimenter taking notes.

During this first day, each participant also performed the scene construction task modelled on that of Hassabis et al. (2007b); see also Maguire et al., 2010). Each participant was tested individually and sat facing the experimenter. The session was digitally recorded. The instructions were explained to the participant and examples were given. During this practice session, the experimenter continued until they were satisfied that the participant had fully understood what was required of them. Scenarios were chosen that would be suitable for school-aged children (e.g. camping, kite-flying); they were neither fantasy-based nor too similar to events that had happened to them in the past. When participants constructed an imagined scene, they rated how similar it was to actual memory or part of a memory on a scale of 1 (nothing like a memory) … 5 (very like a memory). Only those scenes that scored 1 or 2 on this scale were considered for inclusion (mean of final 10 scenarios: controls 1.54; patients 1.19). For each scenario a short description was read aloud by the experimenter from a prepared script (e.g. “Imagine you are by a campfire in the mountains”) and the participant was to close their eyes and vividly imagine the situation from the cue and describe it out loud in as much detail as possible. Participants were told not to recall personal memories but create something that was completely new. They were told to verbalise their imagined experience until it came to a natural end or they felt no more details could be given. The experimenter could use general probes to encourage the participants (e.g. “can you see anything else?”), however it was strictly prohibited to introduce any idea, concept, entity or detail that had not already been mentioned by the participant. After each scenario was recorded, participants were asked to rate it according to a number of different criteria (see Section 3). At various points during the trial and before the post-scenario ratings, the experimenter confirmed that the participant was still familiar with the task instructions, the scenario in question, and the scenario that had been created.

On day 2, each participant was asked to recall out loud, one at a time, and in as much detail as possible, each of the ten events from the previous day in response to short verbal cues (e.g. “recall choosing a snack”). Also on day 2 they were required to recall out loud in as much detail as possible the ten imagined scenarios from the day before. All descriptions of imagined events on day 1 and real and imagined events on day 2 were recorded, anonymised and then transcribed (blindly) by a professional transcription company.

### 2.3 Scoring

Scoring is described in the Section 3. As some scores were based on the experimenter's ratings, and the primary experimenter was not blind to participant status, four subjects were selected at random (2 patients and 2 controls; 12% of the data) and their data scored by a second experimenter who was blind to participant identity. Cronbach's Alpha was used to assess inter-rater reliability. To summarise, all experimental measures were above 0.7 (range 0.855–0.921) which is considered to indicate reliability (de Vaus, 2002; Gliem & Gliem, 2003). There were no significant differences in reliability for subjective ratings and content measures.

### 2.4 Statistical analysis

The two groups [patients (*n* = 21) and controls (*n* = 12)] were compared using a range of statistical tests: *t*-tests, analysis of covariance with age as a continuous variable, where appropriate non-parametric Mann–Whitney *U* test, and partial correlations controlling for participant age; a significance threshold of *P* < 0.05 was applied throughout. As described above, there were unequal numbers in the two groups. In order to verify that this did not influence the results, we selected 12 patients at random and compared them with the 12 control participants on the experimental recall measures and the scene construction task. The same pattern of results that we report in the Section 3 was found, and at the same statistical threshold as the main analyses, including the significant correlations with hippocampal volume.

## 3 Results

### 3.1 Recall of autobiographical events

Although the patients’ selective memory problems had been identified on baseline neuropsychological assessments, we first wanted to establish how they would perform on a novel naturalistic autobiographical memory recall task. Having been exposed to events on day 1, recall for these autobiographical episodes was assessed on day 2. Note that one patient, while following the protocol of day 1, was not available to test on day 2 and so recall on day 2 was examined in 20 patients.

Recall of each of the ten autobiographical events was assessed using three criteria (each scoring 1 or 0 points). First, we assessed whether a participant could give an overall description of the event in response to a retrieval cue (e.g. “recall choosing a snack”). In order to assess specific episodic details, two other criteria were considered. Spatiotemporal information about the location and/or time of the event, and specific details of the episode that could not be inferred from the retrieval cue (this included details such as thoughts, actions or dialogues that occurred during the event, which were noted covertly by the experimenter at the time, and occurred in all cases). Finally, a scorer rated how well they felt the details provided by the participant reflected the actual event that had taken place on the previous day. This accuracy rating was between 0 (indicating the construction was completely devoid of details pertaining to the event with no sense of recollection) and 10 (indicating an extremely rich and vivid recollection). Table 1 shows the mean scores on the autobiographical recall measures.

Patients and controls provided similar event descriptions (*F* = 0.76; *P* = 0.39). By contrast, controls were significantly better at recalling the specific information that located an event in time and place (*F* = 4.32; *P* = 0.047). Moreover, patients were significantly poorer than controls at recalling the detailed episodic information that made an event unique (*F* = 5.16; *P* = 0.031). Combined with the significantly poorer accuracy ratings ascribed to their recall (*F* = 6.64; *P* = 0.015), the patients’ impaired recollection of spatiotemporal and episodic details confirms that the children exposed to neonatal hypoxia/ischaemia and concomitant hippocampal damage had a clear deficit in autobiographical event recall.

### 3.2 Scene construction

Having confirmed that autobiographical event memory recall was significantly impaired in the patients, we then examined their ability to imagine fictitious scenarios. Their descriptions of newly constructed experiences created on day 1 were analysed in terms of three components: content, participant ratings and spatial coherence. These components had previously been identified by Hassabis et al. (2007b) when assessing scene construction in healthy and amnesic adults (see also Maguire et al., 2010). A similar scoring protocol was employed here with some adjustments to suit paediatric participants (see Section 2.2). See Table 2 for the mean scores on the scene construction measures.

#### 3.2.1 Content

**Fig. 3:**
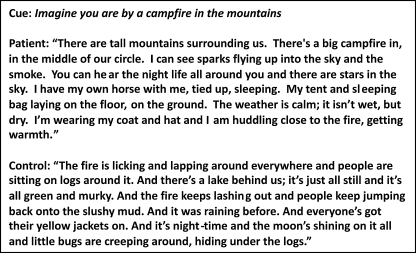
Example transcripts from the scene construction task on day 1.

Each scenario description was segmented into a set of statements. Every statement was then classified as belonging to one of four main categories: the number of entities present (EP), sensory descriptions (SD), thoughts, emotions and actions (TEA) and spatial references (SPA). Hassabis et al. (2007b) found that the production of 7 details per category was an optimal reflection of performance while ensuring that those with more circuitous descriptions were not unfairly advantaged. Thus, the score for each detail category was capped at a maximum of 7 (of note, the results were highly similar when the uncapped data were used). Compared to controls, patients produced a similar amount of each statement type and there were no significant group differences (EP *F* = 2.63, *P* = 0.114; SD *F* = 2.41, *P* = 0.131; TEA *F* = 4.04, *P* = 0.054; SPA *F* = 1.82, *P* = 0.187). See Fig. 3 for example scenario descriptions.

#### 3.2.2 Participant ratings

On completion of each imagined construction, participants were asked to rate their perceived salience from 1 to 5 (1 – ‘couldn’t really see anything’ … 5 – ‘extremely vivid’) to determine how vivid a scene had been in their mind while they were imagining it. They were also asked to rate their sense of presence from 0 to 2 (0 – ‘did not feel like I was there at all’ … 2 – ‘felt like I was really there’). There was no difference between the controls and the patients on either self-rated measure, with both groups feeling they were present within the scenario and that the scene was vivid in their mind (salience *F* = 0.65, *P* = 0.43; presence *F* = 2.69, *P* = 0.11).

#### 3.2.3 Spatial coherence index

As part of the feedback on each scenario, participants were presented with a set of 12 statements each providing a possible qualitative description of the newly constructed experience. Participants were instructed to indicate the statements they felt accurately described their construction. They were free to identify as many or as few statements as they thought appropriate. Of the 12 statements, 4 of them directly suggested a scene was fragmented, 4 directly suggested a scene was coherent, and 4 spoke to other aspects of the scene not necessarily related to its coherence – but which were more likely to be chosen if the scene was coherent. Thus, the number of direct statements relating to coherence/fragmentation was the same. No participant chose all 12 statements, nor did they select directly contradictory statements. One point was awarded for each integrated statement selected and one point taken away for each fragmented statement. This yielded a score between −4 and +8 that was then normalised around zero to give final Spatial Coherence Index score ranging between −6 (totally fragmented) and +6 (completely integrated). Any construction with a negative Spatial Coherence Index was considered to be incoherent and fragmented. Both groups produced coherent constructions, and there was no significant difference between the groups (*F* = 1.23, *P* = 0.28).

Finally, we also assessed the difficulty of scene construction. Each participant was asked to give a rating between 1 and 5 (1 – ‘no difficulty’ … 5 – ‘it was very difficult’) on the completion of each scenario. Controls and patients did not differ (means: controls 1.88; patients 1.93; *F* = 0.25, *P* = 0.62), with both groups finding the task easy to perform.

### 3.3 Recall of imagined scenarios

Having observed a clear difference in the patients between their impaired autobiographical memory recall and their intact ability to imagine fictitious scenarios, we then wondered how they would perform when asked to recall the imagined scenarios. Therefore, as with the autobiographical events experienced on day 1 and tested on day 2, the imagined scenarios constructed on day 1 were also tested on day 2 (see Table 3).

In the first instance, the recalled scenarios were scored for content in the same way as during their initial creation, i.e., in terms of entities present (EP), sensory descriptions (SD), thoughts, emotions and actions (TEA), and spatial references (SPA). The groups did not differ significantly on any category (EP *F* = 2.69, *P* = 0.11; SD *F* = 0.18, *P* = 0.18; TEA *F* = 0.16, *P* = 0.15; SPA *F* = 1.88, *P* = 0.18). On the face of it, it appeared that perhaps the memories of these imagined scenarios were preserved while the autobiographical memories were not. However, our next two measures revealed that this was not the case.

The first measure was a reproducibility rating which was designed to examine the extent to which a recalled scenario reflected that which was imagined in the original construction. For each scenario, the scorer gave a rating between 0 and 10 (0 – ‘no similarity to original construction’ … 10 – ‘virtually identical to the original construction’). The reproducibility rating for the patients was significantly lower (*F* = 4.73, *P* = 0.038) than that for the controls. This suggests that during recall, the patients were not necessarily recalling the original imagined scenario, but were creating a variant of the original or a new scenario. This finding was supported by another measure – a count of the number of elements that truly deviated from the original constructed scene. This showed that the patient group included more new information in the ‘recalled’ scenarios than controls (*F* = 4.05, *P* = 0.05). As to the introduction of new material when recalling autobiographical events, the difference between the group scores only approached statistical significance (*F* = 3.48; *P* = 0.072), suggesting that generation of new material was specific to scene construction.

### 3.4 Relationships between the variables

In the patient group we also examined whether there were any correlations between performance on our autobiographical memory test and scores on our scene construction task, and hippocampal volumes and the standardized memory quotient (MQ, Children's Memory Scale). Mean hippocampal volumes in the patients correlated positively with measures from our autobiographical memory recall task – event description scores (right hippocampus *r* = 0.65, *P* = 0.002; left hippocampus *r* = 0.55, *P* = 0.012), and accuracy ratings (right hippocampus *r* = 0.52, *P* = 0.018; left hippocampus *r* = 0.46, *P* = 0.043). Thus, the poorer the autobiographical memory, the smaller the hippocampal volume. There was no significant relationship between scene construction scores and hippocampal volumes, which is not surprising, given that patients were unimpaired on this task and variance was low.

Measures from our autobiographical memory test correlated positively with the MQ (location/time *r* = 0.47, *P* < 0.045; accuracy rating *r* = 0.55, *P* < 0.014). Similarly, the reproducibility score from the ‘recall’ of imagined scenarios on day 2 also correlated with the MQ score (*r* = 0.53, *P* < 0.021). Thus the higher the MQ, the better the score for recall of real-world events and for recall of imagined scenarios, demonstrating consistency across the different memory measures.

There were no significant correlations between our autobiographical memory measures and performance on the scene construction task. Again, this is not surprising, given that patients were significantly impaired on the former, but were unimpaired on the latter.

## 4 Discussion

While there has been a good deal of research on autobiographical memory in adults (Cabeza & St. Jacques, 2007; Maguire, 2001; Spreng et al., 2009; Svoboda et al., 2006), much less attention has been focused on autobiographical memory in school-aged children (Bauer et al., 2007; Peterson, Grant, & Boland, 2005; Picard et al., 2010; Piolino et al., 2007; Van Abbema & Bauer, 2005), despite its importance for development and subsequent independent living. Here we devised a novel naturalistic autobiographical memory task and found that school-aged children (mean age of 12 years) could recall the events in detail, including spatiotemporal information and specific episodic details. This was in contrast to a group of children with neonatal hypoxia/ischaemia and bilateral hippocampal volume reduction in whom recall was impaired, with specific details of the episodes lost.

While there is a relatively limited literature on autobiographical memory in school-aged children, even less is known formally about their ability to imagine fictitious experiences, despite its clear importance in planning future actions. We found that healthy school-aged children could construct scenarios which were rich, vivid and spatially coherent. Interestingly, despite having a significant autobiographical memory deficit, the children with hippocampal damage were unimpaired at constructing imagined experiences. This is in clear contrast to patients with adult-onset hippocampal damage and amnesia, who are typically impaired at scene construction (Hassabis et al., 2007b).

Our novel test, permitting the naturalistic experiencing of real-world events in an incidental fashion, and their subsequent probing in a surprise memory test allowed us to confirm that by (mean) age 12, recall of recent autobiographical memories is well developed. While severe hypoxia/ischaemia and concomitant hippocampal damage in children still permits the generation of an overall description of an autobiographical event, this lacks a spatiotemporal context and the specific details that make it truly episodic and precisely accurate. This is additional evidence that the hippocampus plays a crucial role in episodic recollection (Maguire, 2001; Mishkin, Vargha-Khadem, & Gadian, 1998; Nadel & Moscovitch, 1997; Tulving, 2002; Vargha-Khadem et al., 2003). Our finding of significant correlations between hippocampal volume and measures from our autobiographical memory task, with poorer memory associated with decreased hippocampal volume, underscores this further. The performance of the children with hypoxia/ischaemia was in fact similar to younger healthy children reported by Bauer et al. (2007). They found that between the ages of 7 and 10 years gist or fact-based information predominated during recall of personal events, with few details pertaining to the spatiotemporal context. This suggests that if episodic recollection emerges along a trajectory, then early hippocampal damage interferes with this development and leads to a plateauing of this ability before the onset of adolescence.

It is all the more surprising, therefore, that the children with hippocampal damage and impaired memory for personal episodes were able to imagine fictitious experiences. Moreover, when asked to recall previously created scenarios, they in fact created essentially new constructions, thus eschewing their memory recall problems, instead relying on their intact ability to construct. This ability could help the children to ‘fill-in’ the gaps in their memory and may make it more difficult to detect the extent of their memory problems in daily life. Thus in a large group of children who had experienced neonatal hypoxia/ischaemia with concomitant hippocampal damage, scene construction appears unimpaired. This accords with the previous case report of patient Jon, with developmental amnesia, who had a similar profile of memory impairment and intact scene construction (Maguire et al., 2010). We therefore conclude that intact scene construction is a consistent and reliable feature of bilateral hippocampal damage sustained in childhood, and developmental amnesia. This is clearly different to patients with hippocampal damage and amnesia acquired in adulthood, where in the main they are significantly impaired at imagining fictitious experiences. As noted earlier, findings in one DA patient, HC (Kwan et al., 2010) seem to run contrary to the other 22 patients (Jon and the current paediatric patients), and work is currently underway to examine the anomalous finding in this patient further (E.A. Maguire personal communication).

The question that naturally arises is what underpins the patients’ intact scene construction? Jon described his ability to construct scenes as something that he had worked on over the years (Maguire et al., 2010). Thus it could be that the patients were using strategies to enhance their ability, or compensatory strategies to circumvent their problem. We think it unlikely, however, that the children (some of whom were as young as 9 years old) were engaged in active or conscious strategies, as none of them declared this when probed.

The children, as in previously documented cases of developmental amnesia, had developed IQs in the normal range. This shows that their semantic knowledge base was intact, and perhaps this was sufficient to permit successful scene construction. The fact that the children were able to acquire semantic knowledge in the face of significant hippocampal damage sustained very early in life suggests there is a mechanism for such learning that is not solely dependent on episodic/autobiographical memory. It may be that this mechanism is also able to sustain scene construction, although the nature of such a mechanism is still debated (Baddeley, Vargha-Khadem, & Mishkin, 2001; Mishkin et al., 1998; Squire & Zola, 1998; Tulving & Markowitsch, 1998). If this is the case, then it will be important in future studies to ascertain how more semantically based scene construction can be differentiated from scene construction that involves true visualisation. Interestingly, this distinction can and has been readily made by adult patients with amnesia, who are very clear that they cannot visualise a scene, even though they are able to produce semantically relevant content (Hassabis et al., 2007b). Given that DA patients sustained such early hippocampal damage, and may never have known what it is like to truly imagine a scene or event, then in the absence of any comparator, it is perhaps not surprising that they consider their scene constructions to be coherent.

While semantic memory may be the basis of intact scene construction in our paediatric patients, Maguire et al. (2010) suggested that Jon's ability to construct imagined scenarios may be supported by some functioning of his residual hippocampal tissue. Although his hippocampi are reduced in volume by 50%, they were nevertheless activated during fMRI when he recalled some of the few autobiographical memories that he retains. Interestingly, the one patient with adult-acquired hippocampal damage and amnesia that has intact scene construction (P01 – Hassabis et al., 2007b, also known as KN – Aggleton et al., 2005; McKenna and Gerhand, 2002), showed fMRI activity in the residual tissue of his right hippocampus during a memory task. It is therefore possible that the children with hippocampal damage in the present study retained some functionality in their residual hippocampal tissue which was sufficient to support scene construction, but not enough for accurate and detailed autobiographical memory.

In this study we have described the effect of early hippocampal damage on autobiographical memory and scene construction in children. It is clear that future studies assessing patients such as these [and indeed Jon (Maguire et al., 2010) and P01 (Hassabis et al., 2007b)] should involve fMRI during scene construction in order to establish if residual hippocampal tissue is active, whether additional brain areas are recruited or up-regulated, or a completely different set of brain regions is engaged compared with controls. To our knowledge, there are no published fMRI studies of autobiographical memory/imagining fictitious experiences in children with hippocampal pathology, or in fact any involving healthy children. This is surprising, given that understanding how such critical cognitive functions develop in the first place may provide key insights into their functional neuroanatomy, and we urge the deployment of fMRI in this developmental context.

## Figures and Tables

**Table 1:** Recall of autobiographical events.

Measure	Mean (SD)	
	Patients (*n* = 20)	Controls (*n* = 12)
Event description	9.05 (1.64)	9.58 (0.67)
Location/time	6.10 (1.94)*	7.67 (2.71)
Episodic details	3.70 (2.66)*	5.83 (2.66)
Scorer accuracy rating	5.95 (2.10)*	7.77 (1.03)

* Significantly different to controls.

**Table 2:** Performance on the scene construction task.

Measure	Mean (SD)	
	Patients (*n* = 21)	Controls (*n* = 12)
Content		
Spatial references	3.35 (1.41)	4.01 (1.44)
Entities present	5.92 (0.92)	6.40 (0.50)
Sensory descriptions	5.34 (1.36)	5.97 (1.46)
Thoughts, emotions, actions	4.53 (1.34)	5.56 (1.28)
Participant ratings		
Sense of presence	1.31 (0.28)	1.16 (0.17)
Perceived salience	4.18 (0.64)	4.00 (0.85)
Spatial coherence index	3.64 (1.44)	4.31 (1.33)

**Table 3:** Recall of imagined scenarios.

Measure	Mean (SD)	
	Patients (*n* = 20)	Controls (*n* = 12)
Content		
Spatial references	2.12 (0.95)	2.60 (1.13)
Entities present	4.82 (1.45)	5.57 (1.09)
Sensory descriptions	3.29 (1.73)	3.93 (1.14)
Thoughts, emotions, actions	3.1 (1.37)	3.93 (1.74)
Scorer reproducibility rating	5.59 (1.68)*	6.81 (1.06)
New information	0.56 (0.56)*	0.26 (0.24)

* Significantly different to controls.
